# Risk-adjusted monitoring of surgical performance

**DOI:** 10.1371/journal.pone.0200915

**Published:** 2018-08-08

**Authors:** Jianbo Li, Jiancheng Jiang, Xuejun Jiang, Lin Liu

**Affiliations:** 1 School of Mathematics and Statistics, Jiangsu Normal University, Xuzhou, Jiangsu 221116, China; 2 Department of Mathematics and Statistics, University of North Carolina at Charlotte, Charlotte, NC 28223, United States of America; 3 Department of Mathematics, Southern University of Science and Technology, Shenzhen, Guangdong 518055, China; 4 The Research Center of Higher Education, Jiangsu Normal University, Xuzhou, Jiangsu 221116, China; University of the Chinese Academy of Sciences, CHINA

## Abstract

We propose a nonparametric risk-adjusted cumulative sum chart to monitor surgical outcomes for patients with different risks of post-operative mortality due to risk factors that exist before the surgery. Using varying-coefficient logistic regression models, we accomplish the risk adjustment. Unknown coefficient functions are estimated by global polynomial spline approximation based on the maximum likelihood principle. We suggest a bisection minimization approach and a bootstrap method to determine the chart testing limit value. Compared with the previous (parametric) risk-adjusted cumulative sum chart, a major advantage of our method is that the morality rate can be modeled more flexibly by related covariates, which significantly enhances the monitoring efficiency. Simulations demonstrate nice performance of our proposed procedure. An application to a UK cardiac surgery dataset illustrates the use of our methodology.

## Introduction

Monitoring surgical outcomes of clinical trials is a critical task for doctors to timely detect patient deterioration. However, this task becomes complicated for assessing and online monitoring surgical performance. It is known that the surgical performance changes as patient characteristics, such as age, weight, blood pressure, and pulmonary status, varies. Therefore, assessing and monitoring surgical performance should be adjusted according to the patient’s characteristics existing prior to the surgery. This process is the so-called risk adjustment.

There are several methods for monitoring outcomes of surgery. Examples include the Shewhart chart, the sequential probability ratio test, the exponential weighted moving average (EWMA), and the cumulative sum control chart (CUSUM). Recent studies have suggested to use such schemes to monitor the performance of clinical practitioners, including surgical and general practitioners. Among all the control charts, CUSUM has received much attention because of its simple formulation, intuitive representation, and capability to detect small persistent changes, since it was originally proposed [1]. CUSUM was first applied [2] for surgical performance monitoring. Then it was used [3, 4] for monitoring pediatric cardiac surgeries, among others. For an overview, see references [5–7].

Consider a cardiac surgery example. During 1992–1998, a UK center for cardiac surgery facilitated 6994 cardiac operations including 5212 males and recorded information such as date, surgeon, and Parsonnet score formed by age, gender, hypertension and diabetic status, renal function and left ventricular mass [8]. The age varies between 11 and 99 with mean 62.5, median 64 and standard deviation 11. According to the records, 461 patients died within 30 days of surgery, corresponding to a mortality rate of 6.6%. Hence, it is demanded to assess and monitor the surgical performance in order to help doctors reduce the mortality rate and adjust the surgical plan in subsequent operations. One can use the CUSUM charts in [2–4] for this task. However, there is no patients’ preoperative risk considered in these works. Such straightforward applications might lead to a biased assessment of surgical performance because of heterogeneity of patients. As indicated in [9], without risk adjustment for heterogeneity among patients, the control chart shows outcomes confounding with the preexisting risk factors. Hence, it is necessary to develop a risk-adjusted (RA) CUSUM for this example.

There are various works on the RA CUSUM in the literature using two classes of models, respectively for discrete and continous outcomes. For discrete outcomes, examples include the RA CUSUM charts for Down’s syndrome which adjusted the risk of the age of mother using logistic regression [10], for shoulder surgery which adjusted for patients’ rehabilitation conditions [11], and for binary cardiac surgical outcomes which adjusted the risk using a likelihood-based scoring method [12]. In a discussion paper [13], advantages and disadvantages of various control charts including the RA CUSUM were discussed for monitoring health-care and public-health surveillance. The incremental advantage of RA CUSUM was further assessed for coronary bypass outcomes [14] using the procedure in [8]. The RA CUSUM was also investigated in [15] for binary outcomes using the logistic regression and the Bayesian method. For continuous outcomes, examples include the RA CUSUMs for survival times using the Cox model [16] and the accelerated failure model [9], among others. However, these cusum charts are all risk adjusted based on parametric models.

To adjust the risk factors in the UK cardiac surgery example, a linear logistic regression was used to model the relationship between the surgical outcome and the Parsonnet score [8]. Let *t* = 1, 2, … be the indexes for the patients undergoing surgery in time order. The RA CUSUM chart is used to monitor their outcomes. Let *Y*_*t*_ = 1 if patient *t* dies and *Y*_*t*_ = 0 otherwise. Given the *t*-th patient’s outcome *Y*_*t*_ and Parsonnet score *P*_*t*_, the model takes the following form:

$$\begin{array}{c} \text{logit}\left(p_{t}\left(\theta \right)\right)=\theta_{0}+P_{t}\theta_{1}, \end{array}$$ (1)

where *p*_*t*_ is the mortality rate defined as *P*(*Y*_*t*_ = 1|*P*_*t*_), and logit(*t*) = log(*t*/(1 − *t*)) is the logit function. Then the conditional probability mass function of *Y*_*t*_ given *P*_*t*_ is $f\left(y,\theta \right)=p_{t}{\left(\theta \right)}^{Y_{t}}{\left[1-p_{t}\left(\theta \right)\right]}^{1-Y_{t}},$ and the mortality odds of failure for patient *t* is *p*_*t*_/(1 − *p*_*t*_). Since different patients have different baseline risk levels, one needs to monitor the change of odds ratio of patients. Let *R*_*t*_ be the mortality odds ratio of patient *t*. It is interesting to use the RA CUSUM to sequentially test [8]:

$$H_{0}:\;R_{t}=R_{0}\;\;\text{versus}\;\;R_{t}=R_{A},$$

where *R*_0_ is typically the mortality odds determined by the current process performance, and *R*_1_ corresponds to an inferior performance. Mathematically, it can be verified that, the probability of failure *P*(*Y*_*t*_ = 1|*P*_*t*_) equals *R*_0_*p*_*t*_/(1 − *p*_*t*_ + *R*_0_*p*_*t*_) and *R*_*A*_*p*_*t*_/(1 − *p*_*t*_ + *R*_*A*_*p*_*t*_) under *H*_0_ and *H*_*A*_, respectively. Hence, the log likelihood ratio for patient *t* is [8]

$$\begin{array}{c} W_{t}=\{\begin{array}{cc} \log\{\left[\left(1-p_{t}+R_{0}p_{t}\right)R_{A}\right]/\left[\left(1-p_{t}+R_{A}p_{t}\right)R_{0}\right]\} & \text{if}\;\;Y_{t}=1,\\ \log[\left(1-p_{t}+R_{0}p_{t}\right)/\left(1-p_{t}+R_{A}p_{t}\right)] & \text{if}\;\;Y_{t}=0. \end{array} \end{array}$$ (2)

Then the RA CUSUM statistics are defined as

$$\begin{array}{c} Z_{t}=\max\left(0,Z_{t-1}+W_{t}\right),\;\;t=1,2,\cdots , \end{array}$$ (3)

where *Z*_0_ = 0. When the value of *Z*_*t*_ exceeds a certain threshold value *h*, a change in value has been found, and an alarm is signaled.

The model parameter *θ* and hence *p*_*t*_ are estimated by maximizing the likelihood of the in-control dataset ${\left\{Y_{t},P_{t}\right\}}_{t=1}^{n_{1}}.$ Specifically, *θ* is estimated by maximizing the likelihood:

$$\prod_{t=1}^{n_{1}}p_{t}{\left(\theta \right)}^{Y_{t}}{\left[1-p_{t}\left(\theta \right)\right]}^{1-Y_{t}}.$$

The threshold *h* is usually decided by the average run length (ARL). For detail see the monitoring algorithm to be introduced later. Since {*Z*_*t*_} is decided by the underlying process of *p*_*t*_ in model (1), success of the RA CUSUM depends on if the model of *p*_*t*_ is appropriate.

If the null hypothesis is rejected, it indicates a significant increase in the mortality rate. By using this method, the Parsonnet score was found to significantly affect the mortality rate, and it was also claimed [8] that this procedure could detect changes in surgical performance earlier than the non-adjusted CUSUM. However, this approach is based on the assumption in model (1) that the log odds ratio of mortality rate is a linear function of the Parsonnet score. This may create modeling bias if the underlying relationship is nonlinear. In particular, the modeling bias becomes more serious when there are interaction effects among the patient characteristics.

To alleviate the modeling bias problem and to cope with possible interaction effect among the patient characteristics, we propose the following varying-coefficient logistic regression (VCLR) model:

$$\begin{array}{c} \text{logit}\left(p_{t}\right)=\mu +X_{t}^{\prime }\beta \left(U_{t}\right), \end{array}$$ (4)

where *X*_*t*_ = (*X*_1*t*_, *X*_2*t*_, ⋯, *X*_*qt*_)′, *p*_*t*_ = P(*Y*_*t*_ = 1|*X*_*t*_, *U*_*t*_), *μ* is the intercept term, *U*_*t*_ is a random variable, for example, any entry of *X*_*t*_, and *β*(*u*) = (*β*_1_(*u*), *β*_2_(*u*), ⋯, *β*_*q*_(*u*))′ with *β*_*i*_(*u*) being unknown functions. When *β*(*u*) is a constant vector, model (4) reduces to logistic linear regression which includes model (1). Model (4) allows us to model nonlinear relationship between *p*_*t*_ and *X*_*t*_. If *U*_*t*_ is one entry of *X*_*t*_, it captures nonlinear interaction among *X*_*t*_. For the UK cardiac surgery example, we take the *t*-th patient’s age as *U*_*t*_ and Parsonet score *P*_*t*_ as *X*_*t*_. It is believed that the effect of a patient’s Parsonet score depends on the age, and it is interesting to investigate if there is a nonlinear interaction effect between the Parsonet score and age. Therefore, model (4) can be used to fit the UK cardiac surgery dataset.

We estimate *β*(⋅) by the maximum likelihood principle with a polynomial splines approximation in the next section. Then we adjust CUSUM statistics *Z*_*t*_ in Eq (3) for monitoring the change of odds ratio of patients. Since we use nonparametric model (4) to adjust the risk, our proposed RA CUSUM is a nonparametric RA method. We propose a bisection search algorithm and a bootstrap method to determine the nonparametric risk-adjusted CUSUM chart limit value. Through simulations and a real data example, we illustrate nice performance and the use of the proposed methodology.

## Monitoring procedure

### Polynomial spline approximation

Global polynomial spline approximation has become a popular tool in nonparametric smoothing. It has advantages of nice finite sample performance and fast implementation. For function *β*(⋅), it can be approximated by a linear combination of the basis splines (B-splines).

Assume that random variable *U*_*t*_ has finite support [*a*, *b*]. Let *r* be the degree of B spline polynomial, and let *ξ*_1_ = *ξ*_2_ = ⋯ = *ξ*_*r*_ = *a* < *ξ*_*r*+1_ < *ξ*_*r*+2_ < ⋯ < *ξ*_*r*+*N*_ < *b* = *ξ*_*r*+*N*+1_ = *ξ*_*r*+*N*+2_ = ⋯ = *ξ*_2*r*+*N*_ be the knots for the B spline approximation, where $N=O(n_{1}^{v})$ with 0 < *v* < 0.5 such that $\max_{1\leq k\leq N+1}\left\{\right|\xi_{r+k}-\xi_{r+k-1}\left|\right\}=O\left(n_{1}^{-v}\right)$. Usually we call ${\left\{\xi_{i}\right\}}_{i=r+1}^{d}$ with *d* = *r* + *N* the inner knot points. The number of inner knots, *N*, is a tuning parameter and can be chosen by cross validation [17] or generalized cross validation (GCV) [18, 19]. We denote by ${\left\{B_{j}\left(u\right)\right\}}_{j=1}^{d}$ the B-spline basis functions based on the knot set ${\left\{\xi_{i}\right\}}_{i=1}^{2r+N}$. Then the B-spline basis functions enjoy the following properties [20]:

*B*_*j*_(*u*) = 0 for *u* ∉ [*ξ*_*j*_, *ξ*_*j*+*r*_];
*B*_*j*_(*u*) > 0 for *x* ∈ [*ξ*_*j*_, *ξ*_*j*+*r*_];
$\sum_{j=1}^{d}B_{j}\left(u\right)=1$ for any *u* ∈ [*a*, *b*] and 0 otherwise.

Consequently, for any 1 ≤ *j* ≤ *d* and any real *u*, we have *B*_*j*_(*u*) ∈ [0, 1]. Given the knots ${\left\{\xi_{i}\right\}}_{i=1}^{2r+N}$, *β*_*i*_(*u*) can be approximated by

$$\begin{array}{c} \beta_{i}\left(u\right)\approx \sum_{k=1}^{d}\theta_{ik}B_{k}\left(u\right)=B{\left(u\right)}^{T}\theta_{i}, \end{array}$$ (5)

where *B*(*u*) = (*B*_1_(*u*), *B*_2_(*u*), ⋯, *B*_*d*_(*u*))′ and *θ*_*i*_ = (*θ*_*i*1_, *θ*_*i*2_, ⋯, *θ*_*id*_)′. Let *V*_*t*_ = (*B*(*U*_*t*_)′ *X*_1*t*_, …, *B*(*U*_*t*_)′ *X*_*qt*_,)′ and $\theta ={\left(\theta_{1}^{\prime },\ldots ,\theta_{q}^{\prime }\right)}^{\prime }$. Then model (4) reduces to

$$\begin{array}{c} \text{logit}\left(p_{t}\right)=\mu +V_{t}^{\prime }\theta . \end{array}$$ (6)

Therefore, the maximum likelihood estimation method can be directly used to fit model (6) with the in-control dataset. This estimation method is standard in nonparametric smoothing [19] and can be implemented via some existing programs, for example **glm** and **bs** in the R software.

### Cusum monitoring

Based on the in-control observations ${\left\{\left(Y_{t},X_{t},U_{t}\right)\right\}}_{t=1}^{n_{1}}$, we obtain $\hat{\theta }={\left({\hat{\theta }}_{1}^{\prime },{\hat{\theta }}_{2}^{\prime },\cdots ,{\hat{\theta }}_{q}^{\prime }\right)}^{\prime }$ and $\hat{\mu }$, the maximum likelihood estimate of the B-spline coefficient *θ* and the general mean *μ* in model (6). Then, the *i*th functional coefficient *β*_*i*_(*u*) can be estimated by ${\hat{\beta }}_{i}\left(u\right)=B{\left(u\right)}^{\prime }{\hat{\theta }}_{i}$. This leads to the estimate of mortality rate *p*_*t*_:

$${\hat{p}}_{t}=\exp\left[\hat{\mu }+X_{t}^{\prime }\hat{\beta }\left(U_{t}\right)\right]{\left\{1+\exp\left[\hat{\mu }+X_{t}^{\prime }\hat{\beta }\left(U_{t}\right)\right]\right\}}^{-1},$$

where $\hat{\beta }\left(u\right)={\left({\hat{\beta }}_{1}{\left(u\right)}^{\prime },{\hat{\beta }}_{2}{\left(u\right)}^{\prime },\cdots ,{\hat{\beta }}_{q}{\left(u\right)}^{\prime }\right)}^{\prime }.$ Using Eq (2) we obtain the estimate of log-likelihood *W*_*t*_, denoted by ${\hat{W}}_{t}$. By Eq (3), we calculate the estimate of *Z*_*t*_, denoted by ${\hat{Z}}_{t}$. Note the random properties of the in-control statistic ${\hat{Z}}_{t}$. Let *h* be the limit value of this testing procedure. If ${\hat{Z}}_{t}>h$, then we conclude our RA CUSUM chart triggers a signal, which indicates that the mortality rate increases.

The limit value *h* plays a critical role in the CUSUM chart monitoring. It is usually determined by virtue of average run length (ARL) in control, denoted by ARL_0_, the expected run steps from the start of process to the time that the signal is triggered. Optimality of CUSUM in terms of ARL was studied in [21, 22]. A better chart has longer ARL_0_ and shorter ARL_1_ (ARL when the process is out-of-control). Hence, given a large enough ARL_0_, the optimal limit value *h* can be determined. Usually, the optimal limit value *h* has no closed form. Thus, we use a numerical search method, bisection, to decide it. This approach needs predetermined upper and lower bounds for *h*.

Let *L* be the run length for the monitoring process from start until a signal is triggered. Then *L* is a random variable, and ARL is its expectation, namely, ARL = *E*(*L*). The calculation of ARL is critical in determining *h*. ARL is a theoretical value, so it should be estimated during monitoring. It is usually not a good idea to get a large in-control sample for estimating the ARL. In the next section we propose a bootstrap resampling method to achieve the goal.

For a given ARL_0_, we can obtain the limit value *h* through the above procedure. Thus, the CUSUM chart in Eq (3) can be used to monitor the surgical process in the follow up. That is, for the *t*-th patient, *Z*_*t*_ > *h* indicates an abnormal observation, i.e., the process is out of control. In such a case, surgeons should check where and why the process becomes out of control. In addition, the monitoring efficiency can be assessed by calculating ARL_1_, similar to that of ARL_0_.

### Monitoring algorithm

Suppose *h* ∈ [*a*, *b*] with *a* > 0. Given ARL_0_, we propose the following the chart monitoring algorithm:

- **Phase I**. Determination of the optimal limit value *h*_*opt*_.
  1. Draw *K* bootstrap samples from the in-control sample. For each bootstrap sample, use the procedure in the previous section to calculate the RA CUSUM chart statistic ${\hat{Z}}_{t}$. Let ${\hat{Z}}_{t}^{*(1)},\ldots ,{\hat{Z}}_{t}^{*(K)}$ be the *N* realized values of ${\hat{Z}}_{t}$ from all bootstrap samples. Denote by $L_{k}\left(h\right)=\inf\left\{t:{\hat{Z}}_{t}^{*(k)}\geq h,t=1,2,\ldots \right\}$ and $\hat{ARL}\left(h\right)=K^{-1}\sum_{k=1}^{K}L_{k}\left(h\right).$
  2. Use the bisection method to decide the value of *h*:
    (i) Set *h* = *a* and calculate the value of $\hat{ARL}\left(h\right)$. We use ${\hat{ARL}}_{01}$ to denote the calculated value. It is required that ${\hat{ARL}}_{01}<ARL_{0}$. Otherwise, it can be done by choosing a smaller value of *a*.
    (ii) Set *h* = *b* and calculate the value of $\hat{ARL}\left(h\right)$. We use ${\hat{ARL}}_{02}$ to denote the calculated value. It is required that ${\hat{ARL}}_{02}>ARL_{0}$. Otherwise, it can be done by choosing a larger value of *b*.
    (iii) Set *h* = (*a* + *b*)/2 and calculate the value of $\hat{ARL}\left(h\right)$. We use ${\hat{ARL}}_{0m}$ to denote the calculated value.
    (iv) Given a positive integer *M*, if ${\hat{ARL}}_{0m}>ARL_{0}$ and $|{\hat{ARL}}_{0m}-ARL_{0}|>M$, set *b* = (*a* + *b*)/2; if ${\hat{ARL}}_{0m}<ARL_{0}$ and $|{\hat{ARL}}_{0m}-ARL_{0}|>M$, set *a* = (*a* + *b*)/2. In general, the pre-assigned positive integer *M* ranges from 2 to 5 in practice, so that the ARL with *h*_*opt*_ quickly approaches to the nominal *ARL*_0_ with a certain error tolerance.
    (v) Repeat steps (i)-(iv) until $|{\hat{ARL}}_{0m}-ARL_{0}|\leq M.$ Then the optimal value can be taken as *h*_*opt*_ = *h*.
- **Phase II**. Monitoring Phase.
  With the optimal value *h*_*opt*_, we calculate the RA CUSUM in Eq (3) based on the estimated varying coefficient logistic regression model (4). Let ${\hat{Z}}_{t}$ be the estimate of CUSUM statistic. When ${\hat{Z}}_{t}>h_{opt}$, a signal is triggered and monitoring is stopped.

In our experience, the bootstrapping-based bisection numerical method for the determination of *h* performs well and stably. Other numerical approaches can be used. For example, one can search proper value on a given grid of points. One can also use a theoretical distribution of ARL to determine *h*. However, one cannot expect that the theoretical method performs stably, since it depends on the assumption of a theoretical distribution. Hence, we use the above method in our numerical study.

The proposed monitoring method can be modified to detect a decrease in the mortality rate odds ratio by the following RA CUSUM chart

$$Z_{t}=\min\left\{0,Z_{t-1}-W_{t}\right\}.$$

The above algorithm can be updated to this decrease monitoring with a signal triggered as ${\hat{Z}}_{t}<-h_{opt}$.

## Numerical studies

In this section, we conduct simulations to demonstrate nice performance of the proposed approach and to use the UK cardiac surgery data to illustrate the use of our RA CUSUM chart.

### Simulation

The objective of our simulations is to compare our approach with that of Steiner et al. in [8]. We conduct 500 simulations. For each simulation, we set sample size *n* = 3000, where the first one-third observations are used as the in-control process with *n*1 = 1000 and ARL_0_ = 900, and the remaining two-third observations are used for the out-of-control process with sample size *n*_2_ = 2000.

We use cubic B splines with *N* inner knots to approximate *β*(*u*). The inner knots are set as equally spaced sample quantiles of ${\left\{U_{i}\right\}}_{i=1}^{n_{1}}$. We regard *N* as tuning parameter, and it is chosen by minimizing the value of ARL_1_. We evaluate the estimator of *β*(⋅) by its mean squared errors (RMSE):

$$RMSE=\{n_{1}^{-1}\sum_{i=1}^{n_{1}}\left|\right|\hat{\beta }\left(u_{i}\right)-\beta \left(u_{i}\right){\left|\right|}^{2}{\}}^{1/2}$$

over a grid point ${\left\{u_{i}\right\}}_{i=1}^{n_{1}}$.

**Example 1** (Varying-coefficient models) We generate ${\left\{\left(Y_{t},X_{t},U_{t}\right)\right\}}_{t=1}^{n}$ from the following varying coefficient logistic regression model:

$$\text{logit}\left(E\left(Y_{t}\right)\right)=\mu +X_{t}\beta \left(U_{t}\right),$$

where *U*_*t*_ ∼ *U*(−0.5, 0.5), *X*_*t*_ is uniformly distributed on the set {0, 1, ⋯, 20}, and *β*(*u*) = 0.5 cos(*πu*). For the in-control process, *μ* = −3, and for the out-of-control process, *μ* = −3 + log *R*_*A*_, where *R*_*A*_ equals to 0.3, 0.5, 0.8, 1.5, 2, 2.5, 3, 3.5, or 4. These values of *R*_*A*_ are used to monitor the decrease (*R*_*A*_ < 1) and increase (*R*_*A*_ > 1) in the mortality rate odds ratio.

**Fig 1:**
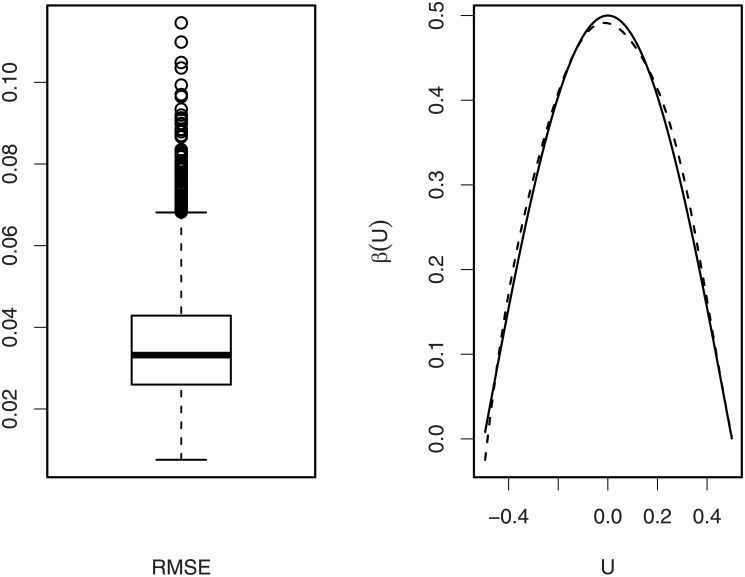
Left panel: The boxplot of RMSEs; right panel: A typical estimate of *β*(*u*), solid—True, dashed—Typical estimate.

Fig 1 shows the boxplot of the RMSE and a typical estimate of *β*(*u*), where the typical estimate corresponds to that with median performance in terms of RMSEs in 500 simulations. It indicates that our estimate is quite close to the true curve. Table 1 summarizes the monitoring results of [8] and ours, where “V-C logistic” stands for the results from the RA CUSUM chart based on the varying coefficient logistic regression model (4), and “Linear Logistic” represents the results from the standard logistic model (1). It is seen that both the mean and variance of ARL_1_ based on the varying coefficient logistic model (4) are significantly smaller than those of ARL_1_ based on the linear logistic model (1). This indicates that our proposed RA CUSUM chart outperforms the RA CUSUM chart of [8].

**Table 1:** Summarized results for Example 1.

*ARL*_1_	V-C Logistic		Linear Logistic	
*R*_*A*_	Mean	Std	Mean	Std
0.3	61.40	34.50	112.12	101.08
0.5	136.91	95.01	247.42	222.77
0.8	499.00	416.55	662.89	531.72
1.5	268.73	212.15	388.06	356.75
2.0	122.75	83.89	176.54	143.94
2.5	77.01	45.76	117.56	94.26
3.0	58.53	35.41	81.50	60.40
3.5	47.67	27.64	66.18	43.93
4.0	39.94	22.30	56.19	37.42

**Fig 2:**
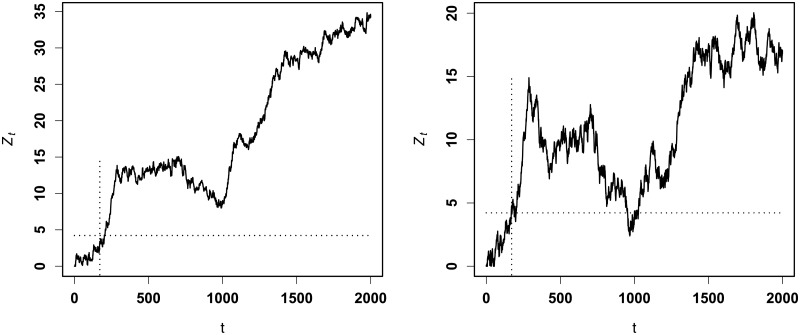
CUSUM charts with *R*_*A*_ = 2.0. Left panel—ours; right panel—[8].

**Fig 3:**
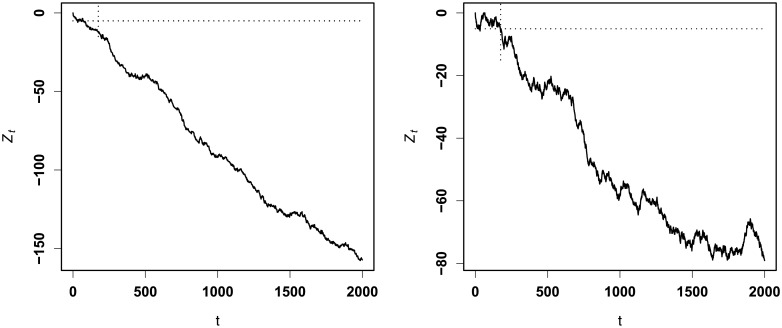
CUSUM charts with *R*_*A*_ = 0.3. Left panel—ours; right panel—[8].

Figs 2 and 3 display the RA CUSUM charts with *R*_*A*_ = 2.0 (increase) and *R*_*A*_ = 0.3 (decrease), respectively. For our RA CUSUM, using the monitoring algorithm, we obtain the values of *h*_*opt*_ as *h*_1_ = 4.2969 for increase monitoring and *h*_2_ = −4.9805 for decrease monitoring. For the RA CUSUM of Steiner et al., the values of *h*_*opt*_ are calculated as *h*_1_ = 4.6876 for increase monitoring and *h*_2_ = −5.7813 for decrease monitoring. Fig 2 shows that our procedure for increase monitoring first triggers a signal at time t = 122, and the linear logistic regression-based CUSUM triggers a signal at time t = 176; for decrease monitoring, the times triggering a signal for our procedure and for that of Steiner et al. are t = 140 and t = 194, respectively. This shows that our procedure triggers a signal much earlier than that of Steiner et al. In addition, we can also conclude from the chart fluctuation that our procedure performs more stably than that of Steiner et al.

**Example 2** (Constant coefficient models) Same as in Example 1, but with *β*(*u*) = 0.5. In this example, since the underlying process has form of model (1), the CUSUM method of Steiner et al. in [8] should work. Since our model (4) contains model (1), as expected, the two RA CUSUM charts perform similarly. The simulated results are reported in Table 2. Since the two procedures have similar values of ARL_1_ according to the mean and standard deviation (Std), the two charts are comparable.

**Table 2:** Summarized results for Example 2.

*ARL*_1_	V-C Logistic		Linear Logistic	
*R*_*A*_	Mean	Std	Mean	Std
0.3	70.40	42.59	71.11	43.38
0.5	151.34	101.30	149.61	99.03
0.8	592.18	482.30	603.92	485.68
1.5	327.46	250.74	336.62	267.37
2.0	151.70	91.92	153.39	94.97
2.5	110.82	74.43	115.83	77.03
3.0	77.68	46.76	78.71	47.00
3.5	64.16	38.96	63.89	36.69
4.0	58.32	32.70	58.01	32.18

### Real data analysis

We here monitor the performance of the UK cardiac surgery using the RA CUSUM charts of [8] and ours. Some details of this dataset were described in the introduction section. For our procedure, we employ the varying-coefficient logistic model

$$\begin{array}{c} \text{logit}\left(p_{t}\right)=\mu +P_{t}\beta \left(Age_{t}\right), \end{array}$$ (7)

which is an extension to the models previously used [8, 14]. Like [8], we treat the data during 1992–1993 as the in-control process and begin monitoring in 1994.

**Fig 4:**
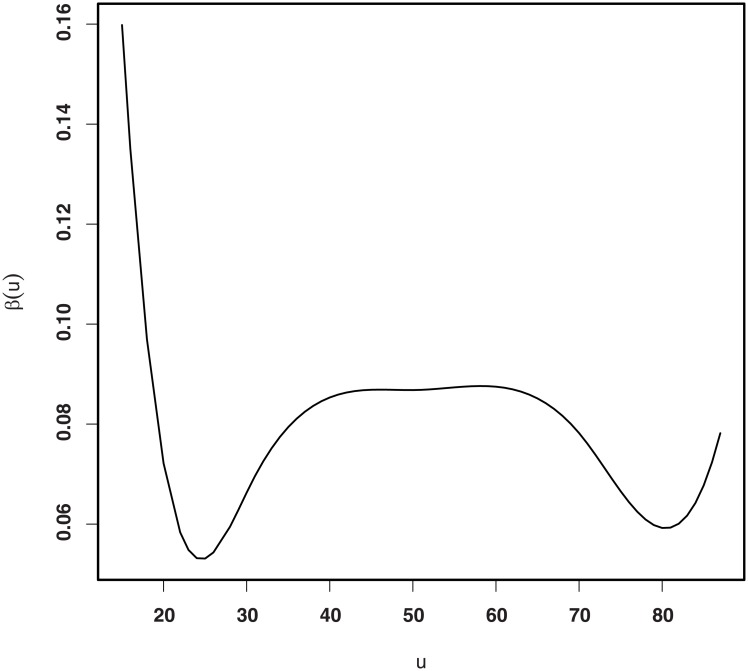
Estimated *β*(⋅) from the in-control process.

We use model (5) to approximate coefficient function *β*(⋅), and the optimal number of knots is calculated as 5. Fig 4 displays the fitted curve of *β*(⋅). It seems that the effect of Parsonnet score nonlinearly depends on Age. In particular, the Parsonnet score strongly correlates with the mortality rate in people aged less than 20 years.

**Fig 5:**
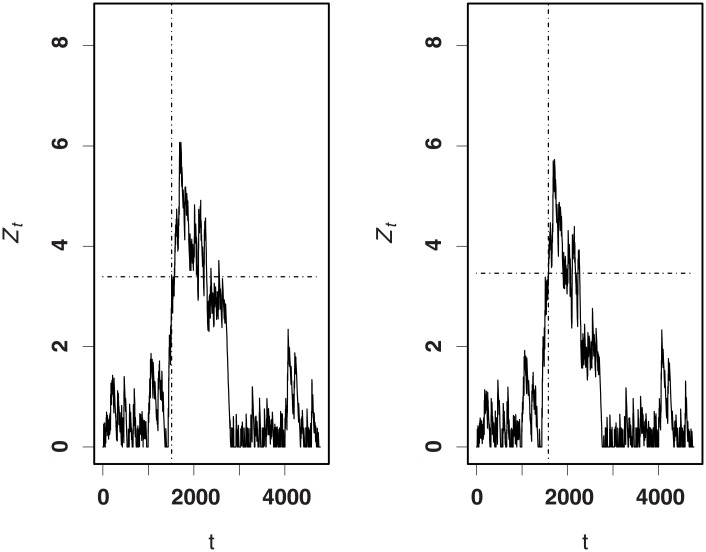
CUSUM charts with *R*_*A*_ = 1.5. Left panel—ours; right panel—[8].

**Fig 6:**
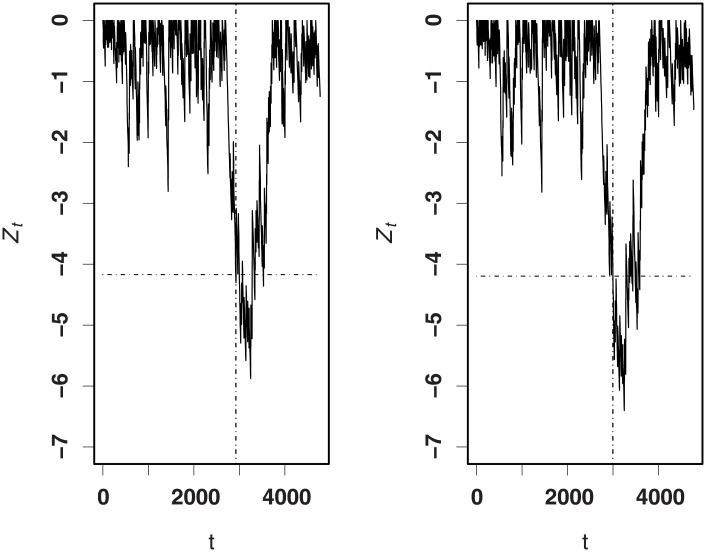
RA CUSUM charts with *R*_*A*_ = 0.5. Left panel—ours; right panel—[8].

To assess the actual performance of the proposed procedure, we compare it with the method of [8] for detecting 1.5 times the odds of death (*R*_0_ = 1, *R*_*A*_ = 1.5) and half of the odds of death (*R*_0_ = 1, *R*_*A*_ = 0.5). By bisection and bootstrapping methods, the values of *h*_*opt*_ for the RA CUSUM charts based on models (4) and (1) are 3.43, 3.50 for the increasing detection and −4.16, −4.22 for the decreasing detection, respectively. Figs 5 and 6 plot the RA CUSUM charts. As shown in the figures, the step number triggering a signal for the increase monitoring is 1572 for our RA CUSUM and 1584 for that of [8]. For the decrease monitoring, the corresponding step numbers are 2921 and 2998, respectively. In all cases, the resulting values of *ARL*_0_ for both method are 2000. These results show that our proposed procedure can detect an abnormal signal much earlier in the decrease monitoring.

## Conclusion

In this paper, we have proposed the nonparametric RA CUSUM chart based on the varying coefficient logistic regression model for monitoring the surgical outcomes. The maximum likelihood and cubic B spline approximation has been used for estimation. The bisection and bootstrap methods have been incorporated to determine the testing limit values. Numerical studies show the advantages of our method over that of [8].

The relationship between the covariates and the mortality rate is usually unknown in applications. Thus, other nonparametric or semiparametric regression may be employed to capture this relationship. The proposed RA monitoring procedure can be extended to other control charts and (or) a mixture of several control charts, such as the charts based on Bayesian approaches [7] and a combination of EWMA and CUSUM charts [23–29].
